# Rapid Sintering of Li_2_O-Nb_2_O_5_-TiO_2_ Solid Solution by Air Pressure Control and Clarification of Its Mechanism

**DOI:** 10.3390/ma11060987

**Published:** 2018-06-11

**Authors:** Hiromi Nakano, Konatsu Kamimoto, Takahisa Yamamoto, Yoshio Furuta

**Affiliations:** 1 Cooperative Research Facility Center, Toyohashi University of Technology, Toyohashi 441-8580, Aichi, Japan; 2 Department of Environmental and Life Sciences, Toyohashi University of Technology, Toyohashi 441-8580, Aichi, Japan; k163413@edu.tut.ac.jp; 3 Department of Materials Design Innovation Engineering, Nagoya University, Nagoya 464-8603, Aichi, Japan; yamataka@numse.nagoya-u.ac.jp; 4 FULL-TECH FURNACE Co., Ltd., Yao 581-0037, Osaka, Japan; president@full-tech.co.jp

**Keywords:** oxide materials, rapid sintering, crystal structure, X-ray diffraction, TEM, HAADF-STEM

## Abstract

We first successfully synthesized Li_1+*x*−*y*_Nb_1−*x*−3*y*_Ti*_x_*_+4*y*_O_3_ (LNT) solid solutions (0.13 ≤ *x* ≤ 0.18, 0 ≤ *y* ≤ 0.06) rapidly at 1373 K for one hour under 0.35 MPa by the controlling of air pressure using an air-pressure control atmosphere furnace. The composition is a formation area of a superstructure for LNT, in which the periodical intergrowth layer was formed in the matrix, and where it can be controlled by Ti content. Therefore, the sintering time depended on Ti content, and annealing was repeated for over 24 h until a homogeneous structure was formed using a conventional electric furnace. We clarified the mechanism of the rapid sintering using various microscale to nanoscale characterization techniques: X-ray diffraction, a scanning electron microscope, a transmission electron microscope (TEM), a Cs-corrected scanning TEM equipped with electron energy-loss spectroscopy, and X-ray absorption fine structure spectroscopy.

## 1. Introduction

In the Li_2_O-Nb_2_O_5_-TiO_2_ system, Li_1+*x*−*y*_Nb_1−*x*−3*y*_Ti*_x_*_+4*y*_O_3_ (0.05 ≤ *x* ≤ 0.3, 0 ≤ *y* ≤ 0.182) (LNT) forms with a superstructure known as the M-phase, which is formed by the periodical insertion of an intergrowth layer in a matrix with a trigonal structure. Since the discovery of the M-phase by Castrejon et al. [1,2], related structures have been investigated [3,4,5,6,7]. On the other hand, a few papers have reported on the Li_2_O-Ta_2_O_5_-TiO_2_ (LTT) system [8]. The Li_1+*x*−*y*_M_1−*x*−3*y*_Ti_*x*+4*y*_O_3_ (LMT, M = Nb or Ta) solid solutions were synthesized with a homogeneous superstructure based on two viewpoints. One is to clarify the self-organized formation area of the superstructure. The second viewpoint is to compare the detailed microstructure between the Nb-solid and Ta-solid solutions [9].

To apply this unique structure as a host material of phosphor, new phosphors have been investigated based on LNT or related structures made by a conventional electric furnace [10]. Recently, we successfully synthesized a red phosphor with high photoluminescence (PL) intensity using an Li_1+*x*_(Ta_1−*z*_Nb*_z_*)_1−*x*_Ti*_x_*O_3_ solid solution as the host material [11,12]. The relationship between the M-phase’s dielectric property and the period of its intergrowth layer has also been studied [13,14]. The oriented LNT bulk ceramics were fabricated by slip casting in a strong magnetic field of 12 T, and we clarified the relationship between their unique qualities and the crystal structure [15].

However, the synthesis of a homogeneous M-phase required treatment at 1373 K for over 24 h, and annealing was repeated several times to complete the reaction in an electric furnace [9]. The sintering time depended on the Ti content, and annealing was repeated for 24–264 h until a homogeneous structure was formed by the insertion of periodical intergrowth layers [9]. Accordingly, a fast sintering technique that uses lower energy is required for the practical application of this material as phosphors and electroceramics. As a rapid sintering technique, the millimeter-wave heating system was used, and it succeeded in synthesizing M-phase at 1273 K for 1 h [7]. This was achieved by a radiation of high-energy (24 GHz) in a high-electric field.

This time, we pioneered a new rapid sintering technique, which uses a simpler furnace that only requires the control of air pressure. The range of pressure is from 0.1 to 0.6. This material formed a periodical structure with high sensitivity on the sintering condition. Therefore, we can check the performance of the new processing route to observe the material’s periodicity and homogeneity by various analytical devices; a scanning electron microscope (SEM), X-ray diffraction (XRD), a transmission electron microscope (TEM), an electron energy-loss spectroscope (EELS), and an X-ray absorption fine structure spectroscope (XAFS). The homogeneity of the material is caused by atomic diffusion, and especially oxygen diffusion in the case of oxide. As a result, the homogeneous M-phase could be synthesized at 1373 K for 1 h by controlling air pressure. A microscale to nanoscale structural analysis of the LNT solid solution was carefully performed. In this paper, we report new knowledge for the rapid sintering of LNT ceramics, in which atomic diffusion was promoted under higher pressure than ordinary pressure.

## 2. Materials and Methods

The starting materials used were Li_2_CO_3_, Nb_2_O_5_, and TiO_2_ (>99.9% grade) to prepare the solid solution of LNT. The compositions of the LNT solid solutions prepared in this work followed the general formula Li_1+*x*−*y*_Nb_1−*x*−3*y*_Ti*_x_*_+4*y*_O_3_ (0.13 ≤ *x* ≤ 0.18, 0 ≤ *y* ≤ 0.06), in which the Ti content was 15, 20, 25, and 30 mol %. These powder specimens were thoroughly mixed with a small amount of ethanol in a planetary ball mill (Pulverisette P-6, Fritsch, Dresden, Germany). The well-mixed materials were subsequently pressed into pellets and heated at 1073 K for 5 h in air as a calcination process. The calcination process is the same as that used in previous experiments [7]. After that, the pellets were sintered using an air-pressure control atmosphere furnace (FULL-TECH FURNACE CO., Ltd., Osaka, Japan) at 1273 K–1373 K for 30 min–1 h under various air pressures. Table 1 shows the relationship between absolute pressure, the gauge pressure of the device, and oxygen partial pressure. Here, [oxygen partial pressure] = [absolute pressure] × [0.206]. [Gauge pressure] = [absolute pressure] − [atmospheric pressure 0.10]. The pressure range of the commercial furnace is from 0.1 to 0.6.

Structural analysis was carried out by XRD using a RINT 2500 (Rigaku Co., Ltd., Tokyo, Japan) operating at 40 kV and 200 mA. The angles were corrected by an external standard method for the calculation of the lattice parameters. Microstructure images were observed with SEM (JST-IT100, JEOL, Tokyo, Japan) operating at 20 kV. TEM images and selected area electron diffraction (SAED) patterns were also observed by 2100 F (JEOL Co., Ltd., Tokyo, Japan) operating at 200 kV and equipped with energy dispersive X-ray spectroscopy (EDS). High-resolution scanning TEM (STEM) images were obtained by a Cs-corrected STEM (ARM-200FC, JEOL Co., Ltd., Tokyo, Japan) operated at 200 kV equipped with an energy filter (GIF Quantum ER system, Gatan Inc., Pleasanton, CA, USA). The specimen for TEM observation was prepared by crushing the material in ethanol, and the thin powders were scooped by a microgrid mesh. The Ti or Nb of XAFS was measured at beam line BL5S1 at the Aichi Synchrotron Center (Seto, Japan) with fluorescence mode or transmission mode at room temperature. The simulation of XAFS was performed using the software Artemis [16].

## 3. Results and Discussion

### 3.1. Relationship between Air Pressure and Grain Growth

**Figure 1:**
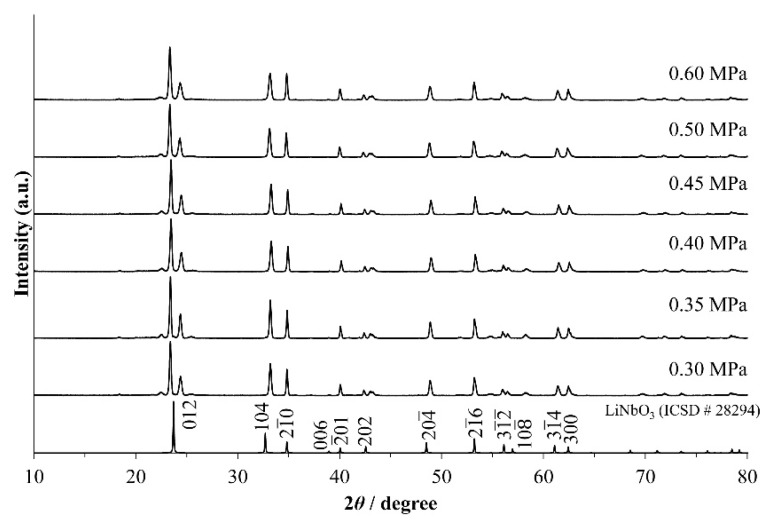
XRD patterns of Li_1+*x*−*y*_Nb_1−*x*−3*y*_Ti*_x_*_+4*y*_O_3_ (LNT) solid solutions with Ti 20 mol % at 1373 K for 30 min under various air pressures.

**Figure 2:**
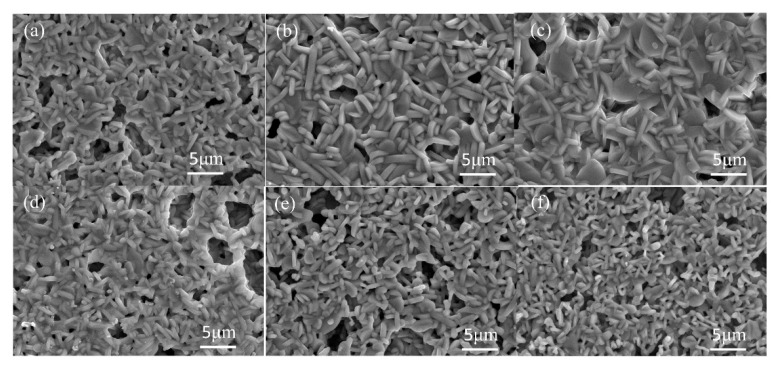
SEM images of LNT solid solutions with Ti 20 mol % at 1373 K for 30 min under various air pressures, (**a**) 0.30 MPa, (**b**) 0.35 MPa, (**c**) 0.40 MPa, (**d**) 0.45 MPa, (**e**) 0.50 MPa, and (**f**) 0.60 MPa.

In order to know the optimal gas pressure for the rapid sintering of LNT ceramics, we sintered LNT with Ti 20 mol % at 1373 K for 30 min at various air pressures from 0.30 MPa to 0.60 MPa. Figure 1 shows the XRD patterns of LNT ceramics with various air pressures. All of the materials show that the satellite reflections appeared around the (012) reflection, which are produced from the superstructure with periodical intergrowth layers. The peak was sharpest at 0.35 MPa and 0.40 MPa, and the peaks of other materials were broader. The grain morphologies and grain sizes were observed by SEM, as shown in Figure 2. There are small pores in the SEM images, and the density of the material does not seem to be so high. Here, we focus on the effect of grain growth, where the densification mechanism should be distinguished from the grain growth mechanism [17].

**Figure 3:**
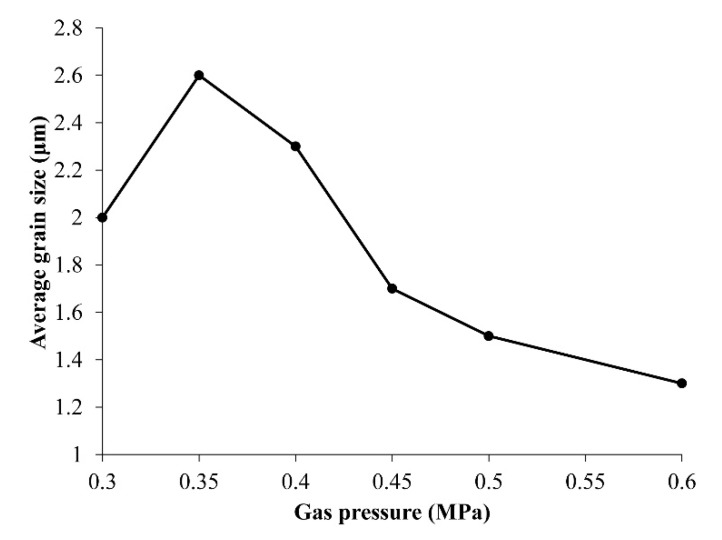
Relationship between average grain size of LNT with Ti 20 mol % and air pressure.

All of the grains were plate-like shapes, although their sizes were obviously different. The relationship between average grain size, which was about 250 grains, and gas pressure revealed that the optimal gas pressure was 0.35 MPa (Figure 3). The grain size should be beneficial for good sintering conditions, because crystallinity improves during grain growth.

**Figure 4:**
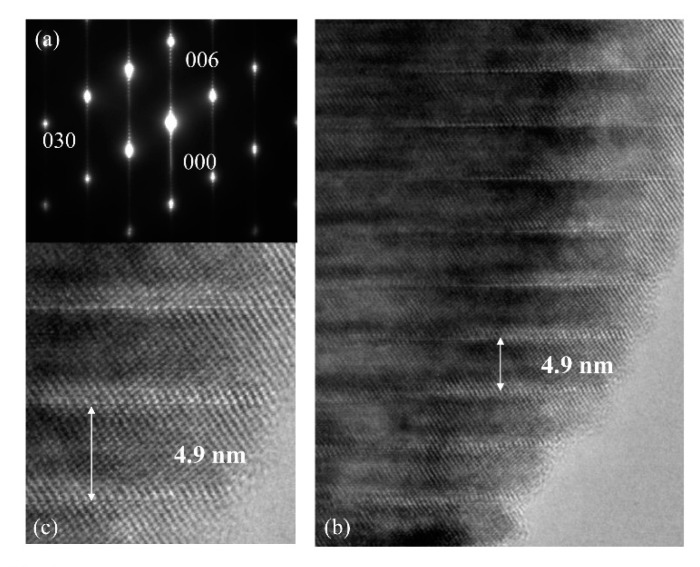
TEM images taken from the [100] axis and the selected area electron diffraction (SAED) pattern of LNT with Ti 20 mol % at 1373 K for 30 min under 0.35 MPa. (**a**) SAED pattern, (**b**) low-TEM image and (**c**) high-TEM image.

It is not so easy to create homogeneous intergrowth layers by Ti distribution using an electric furnace. Smith and West (1992) reported that the homogeneous phase of LNT was synthesized at 1373 K after two to 10 days [1]. As previously reported, we synthesized Li-M-Ti-O (M: Ta, Nb) at 1393 K–1423 K for 72 h to 264 h [9]. We observed the LNT that was sintered under 0.35 MPa by the TEM image taken from the [100] axis and SAED, as shown in Figure 4. The periodical intergrowth layers with 4.9 nm were observed along the *c*-axis, and the period corresponds to 22 times of (006) spacing. The satellite reflections appeared between fundamental reflections, and no streaks were observed. This should be considered amazing as a rapid sintering technique.

### 3.2. Sintering Temperature for Homogeneous Phase of LNT with Various Ti Content

As reported previously, we succeeded in synthesizing LNT at 1273 K for 1 h using millimeter-wave heating [7]. This is a well-known rapid sintering technique for ceramics by the radiation of millimeter-wave (24 GHz). In order to make a comparison between millimeter wave-heating [7] and the present method, the LNT was sintered at 1273 K for 1 h under 0.35 MPa. If the microstructure and the period are different from that of the previous paper, the reaction is insufficient. Therefore, we confirmed the microstructure and the periodicity of the specimens.

**Figure 5:**
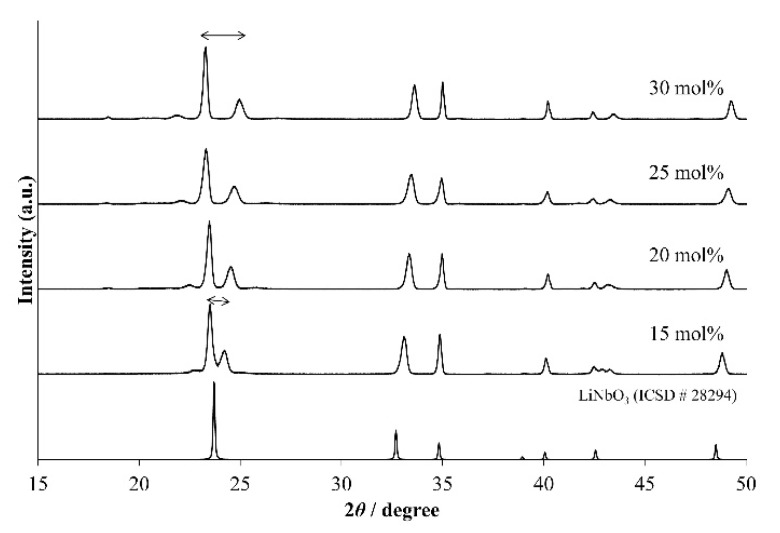
XRD patterns of LNT solid solutions with Ti 15, 20, 25, and 30 mol % at 1273 K for 1 h under 0.35 MPa.

**Figure 6:**
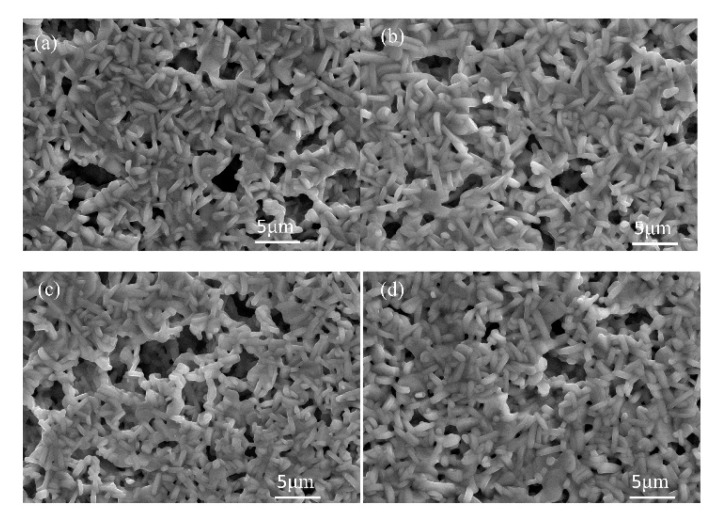
SEM images of LNT with various Ti content at 1273 K for 1 h under 0.35 MPa. (**a**) 15 mol %, (**b**) 20 mol %, (**c**) 25 mol %, and (**d**) 30 mol %.

**Figure 7:**
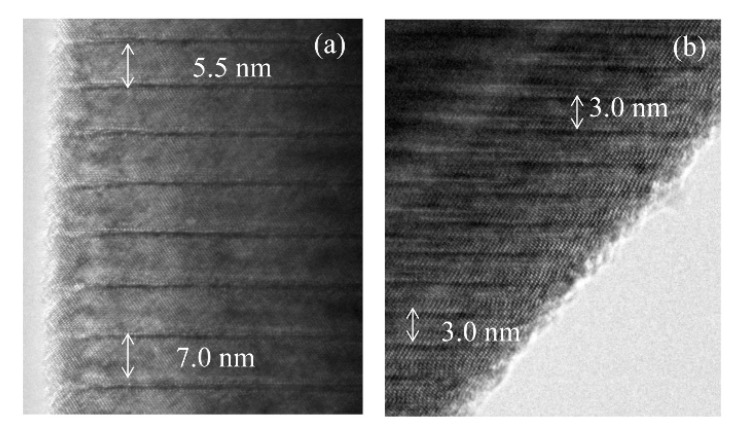
TEM images of LNT with (**a**) 15 mol % and (**b**) 30 mol % at 1237 K for 1 h under 0.35 MPa.

Figure 5 shows the XRD patterns of LNT with various Ti content of 15, 20, 25, and 30 mol % at 1273 K for 1 h under 0.35 MPa. We confirmed the superstructure by detecting the appearance of satellite reflections near the reflection with indices of 012 in the XRD pattern. The results show that the period is controlled by the Ti content, in which the period of satellite reflections was wider with increasing Ti content. However, the peak of LNT with 20 mol % was broader than that seen in Figure 2 at 0.35 MPa. The reason was due to the smaller grain size and the inhomogeneous phase, which can be confirmed by SEM and TEM data. The average size of the grain was 2.2 μm for LNT with 20 mol %. Figure 6 shows SEM images of LNT with various Ti content at 1273 K for 1 h. The average grain size was smaller than that of LNT with 20 mol % synthesized at 1373 K in Figure 2b. Figure 7 shows TEM images of LNT with 15 mol % in (a) and 30 mol % in (b) at 1273 K for 1 h. There are various periods, 5.5 nm or 7 nm, in Figure 7a. In the previous report [7], the period of LNT with 20 mol % is 31 times that of (006); therefore, the period should be 7 nm. On the other hand, the period of LNT with 30 mol % was a relatively homogeneous phase in Figure 7b. The period of 3 nm is 13 times that of (006), and corresponded to our previous result [7]. The intergrowth layers were formed by the diffusion of Ti ions; therefore it is easy to form the homogeneous phase when Ti content is larger [7,9]. These results show that the sintering temperature at 1273 K was insufficient for the formation of the homogeneous phase by controlling the air pressure system.

**Figure 8:**
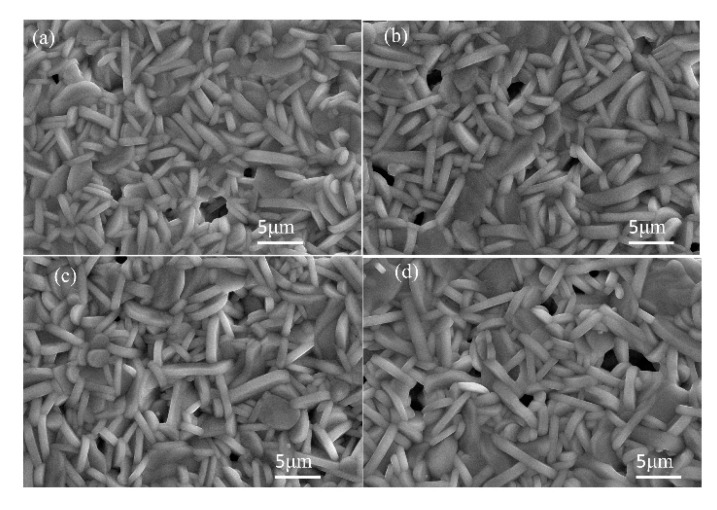
SEM images of LNT with various Ti content at 1373 K for 1 h under 0.35 MPa. (**a**) 15 mol %, (**b**) 20 mol %, (**c**) 25 mol %, and (**d**) 30 mol %.

**Figure 9:**
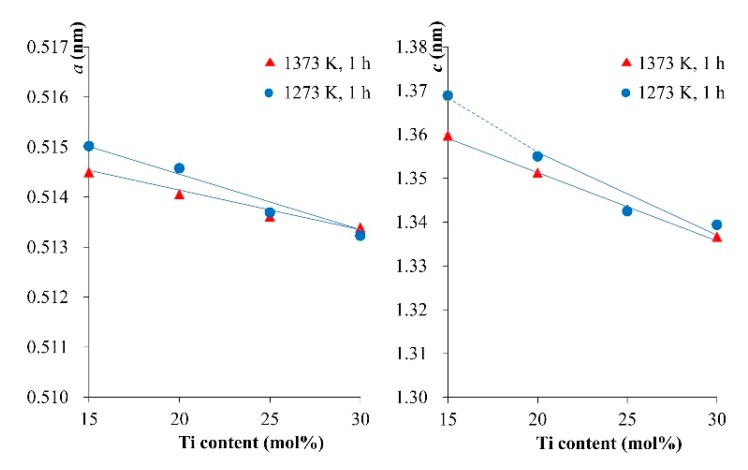
Relationship between lattice constants and Ti content.

Thus, we tried to synthesize the LNT with various Ti content at 1373 K for 1 h under 0.35 MPa. All of the grains increased in size to over 3 μm, which was 1.4 times larger than that of LNT at 1273 K, as shown in Figure 8. The lattice parameters of the LNT with various Ti content were calculated from XRD data and compared between 1273 K and 1373 K, as shown in Figure 9. The results revealed that the cell sizes of *a* and *c* were decreased with increasing Ti content. The LNT sintered at 1373 K decreases more linearly than that of 1273 K. The cause of this is due to the anisotropic nature of the superstructure with strong covalent bonding by insertion of the [Ti_2_O_3_]^2+^ layers [15]. The data indicated that the sintering temperature is suitable for the formation of a homogeneous superstructure of LNT.

### 3.3. Mechanism of Rapid Sintering by Controlling of Air Pressure

**Figure 10:**
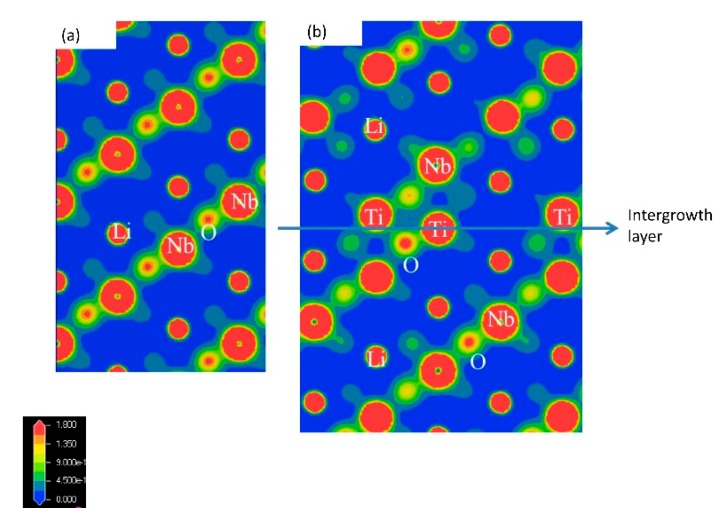
Electron density maps of LiNbO_3_ in (**a**) and Li_9.5_Nb_4.4_Ti_7.1_O_30_ in (**b**) taken from the [100] by the first-principles molecular dynamics simulation [15].

The intergrowth layers of a superstructure are formed by the diffusion of excess Ti ions in the Li-Nb-Ti-O solid solution. In the middle of the time interval of the process, two phases of LiNbO_3_ and a superstructure exist during grain growth in the electric furnace. Therefore, this sintering speed was controlled by Ti content. Figure 10 shows the electron density maps of LiNbO_3_ in (a) and Li_9.5_Nb_4.4_Ti_7.1_O_30_ in (b) taken from the [100] by the first-principles molecular dynamics simulation, as previously reported. We first clarified the anisotropic bonding strength along the *c*-axis in the superstructure [15]. There are Ti ions, O ions, and some vacancies along the intergrowth layer. This was caused from the change in the Ti valence from Ti^4+^ to Ti^3+^ at the intergrowth layer, because the intergrowth layers act as electric conduction paths [15]. The ratio of Ti^3+^ ions were measured by the XAFS spectra of Ti ions in LNT with Ti 20 mol % sintered at 1373 K for 30 min using an electric furnace and an air-pressure control atmosphere furnace under 0.35 MPa. The ratio of Ti^3+^ was 15.2% and 19.6% by electric furnace and air pressure, respectively.

**Figure 11:**
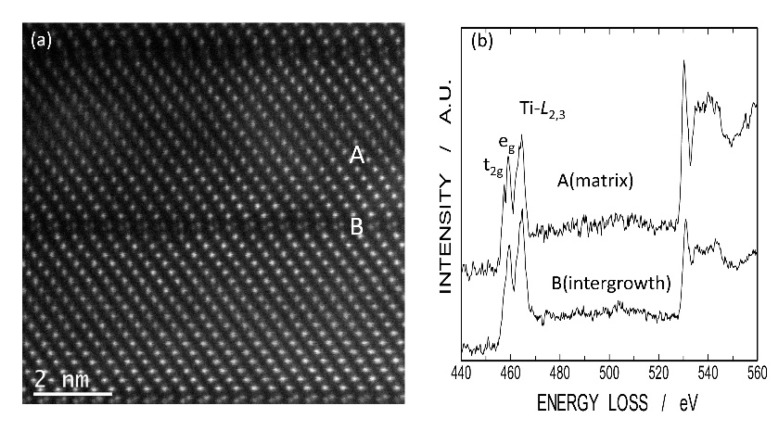
High-angle annular dark field scanning TEM (HAADF-STEM) image of LNT with Ti 20 mol % in (**a**) and electron energy-loss spectroscope (EELS) data for *L*_2,3_-edge of Ti ion at intergrowth layer and matrix in (**b**).

Figure 11 shows a high-angle annular dark field scanning TEM (HAADF-STEM) image of LNT with Ti 20 mol % and EELS of Ti *L*_2,3_ and O K-edges at the intergrowth layer and matrix. There are differences in the fine structures of Ti *L*_2,3_ edges between intergrowth layer (A) and matrix (B). At the intergrowth layer, no sharp splitting of Ti *L*_2,3_-edges was observed, indicating a perturbation in Ti-O octahedral coordination due to high oxygen deficiency [18]. Therefore, the EELS data also showed that Ti^3+^ ions exist at the intergrowth layer.

Until now, there have been few reports about rapid sintering by the controlling of air pressure at a range from 0.3 MPa to 0.6 MPa that corresponds to 0.062 MPa to 0.124 MPa of oxygen partial pressure. Shimanskij et al. (1994) examined oxygen partial pressures from 0.02 MPa to 0.5 MPa for BaTiO_3_, in which the high oxygen partial pressure effectively inhibited grain growth rate during sintering. The paper concluded that the decrease of oxygen vacancies concentration influenced the inhibition of the grain growth [19].

In general, the oxygen vacancy concentration changes by the oxygen pressure in oxides. The oxygen vacancy decreases, and cation vacancy increases with the increasing oxygen partial pressure [20]. Tarento et al. reported (1988) that the dominating defects of the oxygen sublattice were oxygen vacancies at low oxygen partial pressures and oxygen interstitials at high oxygen partial pressures in CoO [21]. Matsunaga et al. interestingly reported that interstitial oxygen can play a more essential role for anisotropic motion in the cell than the presence of O vacancy, which was calculated by the first-principles molecular dynamics simulation in the lanthanum silicate [22].

From our results, we considered that the interstitial oxygen can promote oxygen diffusion with a combination of vacancies along the intergrowth layer, and accordingly, grain growth surprisingly improved by the control of air pressure.

## 4. Conclusions

We first established a rapid sintering technique for Li_1+*x*−*y*_Nb_1−*x*−3*y*_Ti*_x_*_+4*y*_O_3_ solid solutions (0.13 ≤ *x* ≤ 0.18, 0 ≤ *y* ≤ 0.06), which is a simpler system that only requires the control of air pressure in the furnace. The synthesis of a homogeneous M-phase required treatment at 1373 K for over 24 h, and annealing was repeated several times to complete in an electric furnace. 

Using this technique, the homogeneous superstructure of LNT with various Ti content could be synthesized at 1373 K for 1 h under 0.35 MPa. The phase was analyzed carefully by using X-ray diffraction, SEM, TEM, Cs-corrected STEM equipped with an energy filter, and X-ray absorption fine structure spectroscopy. We discovered new knowledge for the rapid sintering of LNT ceramics, in which atomic diffusion was promoted under slightly high air pressure. We concluded that through the control of air pressure, the interstitial oxygen enabled rapid sintering with a combination of vacancies, and that accordingly, grain growth and the distribution of Ti ions improved somewhat surprisingly.

## Figures and Tables

**Table 1:** Relationship between absolute pressure, the gauge pressure of the device, and oxygen partial pressure.

Oxygen Concentration (%)	Absolute Pressure (MPa)	Gauge Pressure (MPa)	Oxygen Partial Pressure (MPa)
20.6	0.10	0.00	0.021
20.6	0.15	0.05	0.031
20.6	0.20	0.10	0.040
20.6	0.25	0.15	0.052
20.6	0.30	0.20	0.062
20.6	0.35	0.25	0.072
20.6	0.40	0.30	0.083
20.6	0.45	0.35	0.093
20.6	0.50	0.40	0.103
20.6	0.55	0.45	0.114
20.6	0.60	0.50	0.124
